# Challenges and efforts in managing AI trustworthiness risks: a state of knowledge

**DOI:** 10.3389/fdata.2024.1381163

**Published:** 2024-05-09

**Authors:** Nineta Polemi, Isabel Praça, Kitty Kioskli, Adrien Bécue

**Affiliations:** ^1^Cybersecurity Lab, University of Piraeus, Piraeus, Greece; ^2^trustilio B.V., Amsterdam, Netherlands; ^3^Research Group on Intelligent Engineering and Computing for Advanced Innovation and Development (GECAD), School of Engineering, Polytechnic of Porto (ISEP/IPP), Porto, Portugal; ^4^School of Computer Science and Electronic Engineering, Institute of Analytics and Data Science (IADS), University of Essex, Colchester, United Kingdom; ^5^Thales Six GTS, Gennevilliers, France

**Keywords:** Artificial Intelligence, trustworthiness, social threats, human factors, cyberpsychology

## Abstract

This paper addresses the critical gaps in existing AI risk management frameworks, emphasizing the neglect of human factors and the absence of metrics for socially related or human threats. Drawing from insights provided by NIST AI RFM and ENISA, the research underscores the need for understanding the limitations of human-AI interaction and the development of ethical and social measurements. The paper explores various dimensions of trustworthiness, covering legislation, AI cyber threat intelligence, and characteristics of AI adversaries. It delves into technical threats and vulnerabilities, including data access, poisoning, and backdoors, highlighting the importance of collaboration between cybersecurity engineers, AI experts, and social-psychology-behavior-ethics professionals. Furthermore, the socio-psychological threats associated with AI integration into society are examined, addressing issues such as bias, misinformation, and privacy erosion. The manuscript proposes a comprehensive approach to AI trustworthiness, combining technical and social mitigation measures, standards, and ongoing research initiatives. Additionally, it introduces innovative defense strategies, such as cyber-social exercises, digital clones, and conversational agents, to enhance understanding of adversary profiles and fortify AI security. The paper concludes with a call for interdisciplinary collaboration, awareness campaigns, and continuous research efforts to create a robust and resilient AI ecosystem aligned with ethical standards and societal expectations.

## 1 Introduction

Artificial Intelligence trustworthiness is a multi-dimensional concept that according to the CEN JTC21 includes cybersecurity, transparency, robustness, accuracy, data quality and governance, human oversight, and record keeping and logging (Newman, 2023). Risk management of trustworthiness implies the identification, analysis, estimation, mitigation of all threats and risks of rising from all these different dimensions where the implementation of a quality management system complements the effective risk management that can be validated through conformity assessment.

Ensuring the trustworthiness of AI systems, involves addressing various risks associated with human factors. Human biases, both implicit and explicit, can inadvertently influence AI algorithms, leading to biased outcomes in decision-making processes. Additionally, human errors during the design, development, and deployment stages can introduce vulnerabilities and compromise the reliability of AI systems. Trust in AI is also contingent on user understanding and acceptance, emphasizing the need for transparent communication about how AI operates and its limitations. Addressing these human-related factors is crucial for enhancing the trustworthiness of AI systems and promoting responsible AI development (Kaplan et al., 2021).

Threats from the different dimensions of trustworthiness are not isolated; they are interrelated and thus the controls and mitigation actions need to be evaluated against their effectiveness in treating all types of risks. For example, human oversights related threats (e.g., bias, luck of transparency/equality, and explain ability) impact cybersecurity (e.g., loss of integrity of data) and vice-versa. The controls needed shall include not only technical but also behavioral, social, cultural, and ethical mitigation actions as it has been clearly mentioned in the AI Act, Article 9, which requires among others that the risk management include the estimation of both technical and human risks.

In this manuscript, our primary goal is to categorize a wide array of threats spanning all dimensions of trustworthiness, leveraging comprehensive classification endeavors, including contributions from prominent entities such as ENISA, NIST (2024), OWASP, MITER, and others. Our aim is to delineate the current challenges in fully comprehending the entire AI threat landscape across all dimensions of trustworthiness and to underscore the research initiatives required to address these challenges. The manuscript will evaluate these standards along with various ETSI and CEN standards and guidelines to gauge their applicability in securing the AI lifecycle and managing AI risks. Notable frameworks, such as NIST AI Risk Management, ENISA Multilayer Framework, and MITER ATLAS, will be scrutinized, and any identified open issues will be highlighted. This manuscript intends to assess the aforementioned efforts, pinpointing existing gaps and open issues, and laying the groundwork for future research and standardization endeavors.

The existing work on AI risk management lacks consideration for human factors and fail to propose metrics for socially related or human threats. As highlighted in the NIST AI RMF (2023) (Appendix C), further research is essential to understand the current limitations of human-AI interaction, an aspect also emphasized by ENISA, which stresses the need for the development of AI ethical and social measurements. Another challenge lies in the selection and implementation of non-technical controls, such as social responsibility. Despite the existence of standards like ISO 26000:2010 and ISO/IEC TR 24368:2022, these are not integrated into the phases of AI risk management. Collaboration is crucial with cybersecurity engineers, AI experts, and professionals from social-psychology-behavior-ethics disciplines to enhance AI risk management methods (EU, 2019). This includes the effective selection of human-related targeted controls. Notably, ongoing EC HE projects (e.g., THEMIS, FAITH) are actively addressing these open issues.

This manuscript is organized as follows: This manuscript is structured as follows: We commence with a comprehensive exploration of the background and ongoing efforts in the dimensions of trustworthiness, encompassing legislation, AI cyber threat intelligence, and the profiles and characteristics of AI adversaries. Subsequently, we delve into the technical threats, vulnerabilities, and attacks associated with AI, followed by an in-depth examination of socio-psychological AI threats, vulnerabilities, and attacks. The discussion then transitions to the management of AI trustworthiness, where we delineate both technical and social mitigation measures. We further elucidate the standards in AI risk management pertaining to trustworthiness, exploring initiatives, frameworks, and notable research projects. Lastly, the manuscript concludes with insights, drawing together key findings and proposing a way forward in the dynamic landscape of AI trustworthiness.

## 2 Background and efforts

### 2.1 Dimensions of trustworthiness

In the rapidly evolving landscape of Artificial Intelligence (AI), the establishment of trust is paramount for widespread adoption and effective integration into various domains. The National Institute of Standards and Technology's (NIST) AI Risk Management Framework (AI RFM) provides a comprehensive set of characteristics that define the trustworthiness of AI systems. This discussion further elaborates on these dimensions, drawing insights from relevant literature and industry practices.

#### 2.1.1 Fit for purpose

The concept of being “fit for purpose” underscores the importance of aligning an AI system's design and capabilities with its intended objectives. This dimension ensures that the system is not only technically proficient but also tailored to meet the specific needs of its users. In the context of AI ethics and design, concepts such as fairness, accountability, and transparency become crucial to achieving a system that is truly fit for its intended purpose (Floridi et al., 2018).

#### 2.1.2 Predictable and dependable

Predictability in AI behavior is a fundamental characteristic that ensures users can anticipate the system's responses and outputs in different situations. Achieving predictability requires transparency in AI algorithms and decision-making processes, enabling users to comprehend and trust the system's operations (Lipton, 2016). Dependability, on the other hand, involves consistent and reliable performance over time, reducing the likelihood of unexpected errors or deviations from established standards (Huang et al., 2020).

#### 2.1.3 Appropriate level of automation

Balancing the level of automation is critical to the ethical and trustworthy deployment of AI. This dimension acknowledges the limitations of AI systems and emphasizes the importance of human oversight, particularly in complex or morally ambiguous situations. Striking this balance ensures that AI augments human capabilities without relinquishing control, promoting responsible and accountable AI practices (Bryson et al., 2017).

The dimensions of trustworthiness in AI, as defined by the NIST AI RFM, encompass being fit for purpose, predictable, dependable, and maintaining an appropriate level of automation. These dimensions provide a robust framework for guiding the development and deployment of AI systems, ensuring they align with ethical considerations and meet the expectations of users and stakeholders in diverse applications.

### 2.2 Legislation

On December 2023, negotiators from the European Union (EU) Parliament and Council reached a preliminary consensus on the EU Artificial Intelligence Act (AI Act). This legislation is most important since it aims to safeguard fundamental rights, democracy, the rule of law, and environmental sustainability from the threats posed by high-risk AI, simultaneously fostering innovation and positioning Europe as a frontrunner in this domain. The regulations set out responsibilities for AI systems based on their potential risks and the magnitude of their influence. The EU AI Act also establishes compulsory guidelines for trustworthy AI, barring specific AI applications that pose “unacceptable risk,” such as those violating fundamental rights, potentially manipulating individuals using subliminal techniques, exploiting vulnerable groups like children, enabling social scoring by public authorities, or allowing remote biometric identification by law enforcement in public spaces. Moreover, the Act sets standards for “high-risk” AI systems concerning data governance, transparency, human oversight, accuracy, and security, with flexibility in implementation but without specifying exact technical solutions. The categorization of high-risk AI depends on its intended use, encompassing eight predetermined areas such as biometric identification, critical infrastructure management, education, employment, access to services, law enforcement, migration control, and democratic processes.

Relevant cybersecurity legislation that apply to AI systems also include: The General Data Protection Regulation (GDPR)-2016; Cybersecurity Act (CSA)-2019, Data Governance Act- DGA applicable from 09/2023; Radio Equipment Directive (RED) applicable from 08/2025; Digital Services Act (DSA)- 11/2022; Critical Entity Resilience Directive; (CER)-11/2022; Network and Information Security Directive 2 (NIS 2)-12/2022; Digital Operational Resilience Act (DORA)-01/2023; Machinery Regulation (MR)-05/2023; Data Act EU-formally adopted-11/2023; Cyber Resilience Act (CRA)-Trilogue started-09/2023. As already mentioned, a political agreement on the AI Act was reached among the European Parliament, Council, and Commission, marking the world's first comprehensive legal framework for AI (AI Act- Trilogue started on 06/2023).

## 3 AI Cyber Threat Intelligence

### 3.1 Integrating human insights and technology

AI Cyber Threat Intelligence (CTI) is essential for analyzing and mitigating vulnerabilities in AI systems. Recent research has detailed cyber-attacker taxonomies and cyber-threat characteristics, identifying adversarial ML threats and various AI Risk Management Frameworks like ENISA, NIST, and MITRE ATTACK. However, these frameworks often overlook the psychological and behavioral profiles of attackers, focusing instead on technical threat assessments without fully considering the cognitive aspects of cybersecurity operators using AI tools.

There's a need for more comprehensive testing that includes cognitive and perceptual dynamics between operators and AI systems, such as task switching, situational awareness, and trust levels. Behavioral change processes, often neglected due to their complexity, are crucial for a deeper understanding of cybersecurity dynamics. Incorporating digital twins could provide dynamic representations of attackers and enhance our understanding of human-AI interactions in cybersecurity.

To advance AI CTI, a socio-technical perspective that includes profiling cyber attackers beyond technical analysis is necessary. Adopting a human-centric approach and cyber-social exercises can help analyze ML attack lifecycles and identify socio-driven vulnerabilities. Developing comprehensive attacker and operator profiles using digital twin technology will allow for more accurate risk assessments. Enhancing human-AI interaction requires investigating behavior-change interventions and applying methodologies like the Unified Theory of Acceptance and Use of Technology (UTAUT) to co-design cybersecurity measures tailored to operators' needs. Exploring the efficiency of human-AI teams in cybersecurity and focusing on Team Decision Making (TDM) processes is also essential.

Standardized AI risk management processes, such as ISO 23894 and the NIST AI 100-1 Lifecycle, provide frameworks for assessing AI system risks throughout their lifecycle. These models and the input from ETSI's AI threat ontology, along with information security standards like the ISO 27000 family, highlight the need for integrated approaches that consider both cybersecurity and AI risks.

Explainable AI (XAI) techniques are crucial for detecting and mitigating biases, monitoring for adversarial attacks, and ensuring the integrity of AI systems throughout their lifecycle. However, integrating XAI into standard risk assessment and adversarial attack detection practices remains a gap. Developing an integrated cybersecurity framework that utilizes XAI throughout the AI lifecycle is necessary. Unifying AI threat knowledge with cybersecurity and privacy insights is crucial for a comprehensive understanding of vulnerabilities and risks. Leveraging XAI to identify potential vulnerabilities and monitor for adversarial ML attacks can enhance risk assessment and control recommendations. Standards under development, such as ISO/IEC/IEEE 24748-7000:2022, are beginning to address these concerns.

Overall, enhancing AI cybersecurity requires a multifaceted approach that includes understanding the profiles and characteristics of AI adversaries, adopting human-centric strategies, and integrating advanced technologies like digital twins and XAI. This approach will lead to more resilient and adaptable security systems, aligning with societal expectations and norms.

### 3.2 Technical threats, vulnerabilities, and attacks

**Figure 1:**
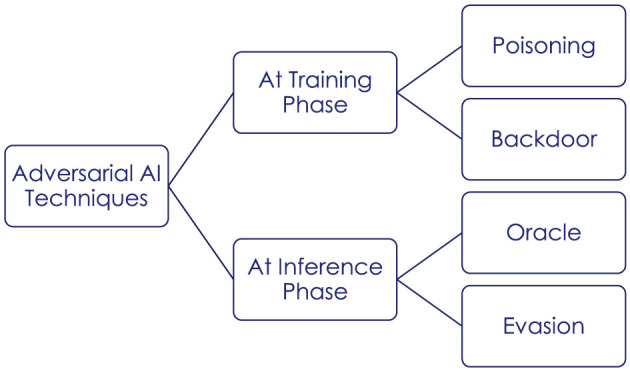
Attacks on AI.

The literature traditionally distinguishes adversarial AI threats based on targets, attack timing, attacker knowledge, and attack consequences (Tabassi et al., 2019). Targets can belong to the physical domain (e.g., sensor input manipulation), in the digital representation (e.g., pre-processed data input), or in the model itself (e.g., a classification model). Attack timing can be at training stage or at inference stage. Attacker knowledge varies from white-box (complete knowledge) to black-box (no knowledge) through diverse shades of gray, depending on attacker knowledge about the model architecture, parameters, training technique and training data. Attack can bare consequences on integrity, availability and confidentiality properties. Figure 1 illustrates the main types of attacks at each stage of the AI lifecycle.

Here under, we provide a summary of known adversarial AI techniques based on these properties.

Data access, poisoning and backdoors are attacks happening at the training stage. With access to training data (e.g., leaked or public), an attacker can create a substitute AI model to use as a testbed for future attack steps (Tabassi et al., 2019). This type of attack requires knowledge of the training data and is therefore a white-box or gray-box attack, depending on the level of knowledge that the attacker has on the model itself. Data access primarily affects the confidentiality, although as a preliminary step to attacks on the model it can have further consequences. Poisoning attacks involve techniques to inject or manipulate training data (Chakraborty et al., 2018). Indirect poisoning occurs before pre-processing and therefore does not require privileges but a good knowledge of the application domain. Direct poisoning occurs after pre-processing and therefore requires access to the training environment. Poisoning attacks generally can affect the integrity and availability of AI models. Unlike data access or poisoning, AI backdoors do not address the physical domain nor its digital representation but the model itself. Side module insertion is the process of adding supplementary nodes to perform hidden tasks in a neural network architecture. Alternatively, deep alteration techniques introduce bias through modification of selected nodes (Aufrant and Hervieu, 2020). Backdoors require complete knowledge and can affect all system properties.

Evasion and oracles are attacks happening at model inference stage. With evasion attacks, an adversary aims to find small input perturbations that cause important changes in the output (Biggio and Roli, 2018). Gradient-based techniques are widely used to cause misclassification in computer vision systems. Gradient-free attacks are possible alternatives in the case the target AI is using gradient-masking techniques (Carlini et al., 2019). Evasion attacks can be carried with limited preliminary knowledge, although black-box attacks will typically require many trial and error iterations. Evasion attacks can be targeted (aiming to make the model fail on specific crafted inputs) or untargeted (aiming to reduce overall system performance). Depending on application cases, they can affect system integrity, or availability (Barreno et al., 2006). In oracle attacks, an adversary collects model outputs and available information to infer characteristics about the model or training data (Papernot et al., 2018). In the case of membership inference, the attacker verifies if a given data input was used in the model training dataset (Shokri et al., 2017). In the case of inversion attacks, he aims to reconstruct training data. In the case of extraction attacks, he aims to reverse-engineer the model. Oracle attacks are a good escalator from black box to gray and white-box knowledge levels (Papernot et al., 2018). They affect the confidentiality of AI model and data.

### 3.3 Socio-psychological AI threats, vulnerabilities, and attacks

**Figure 2:**
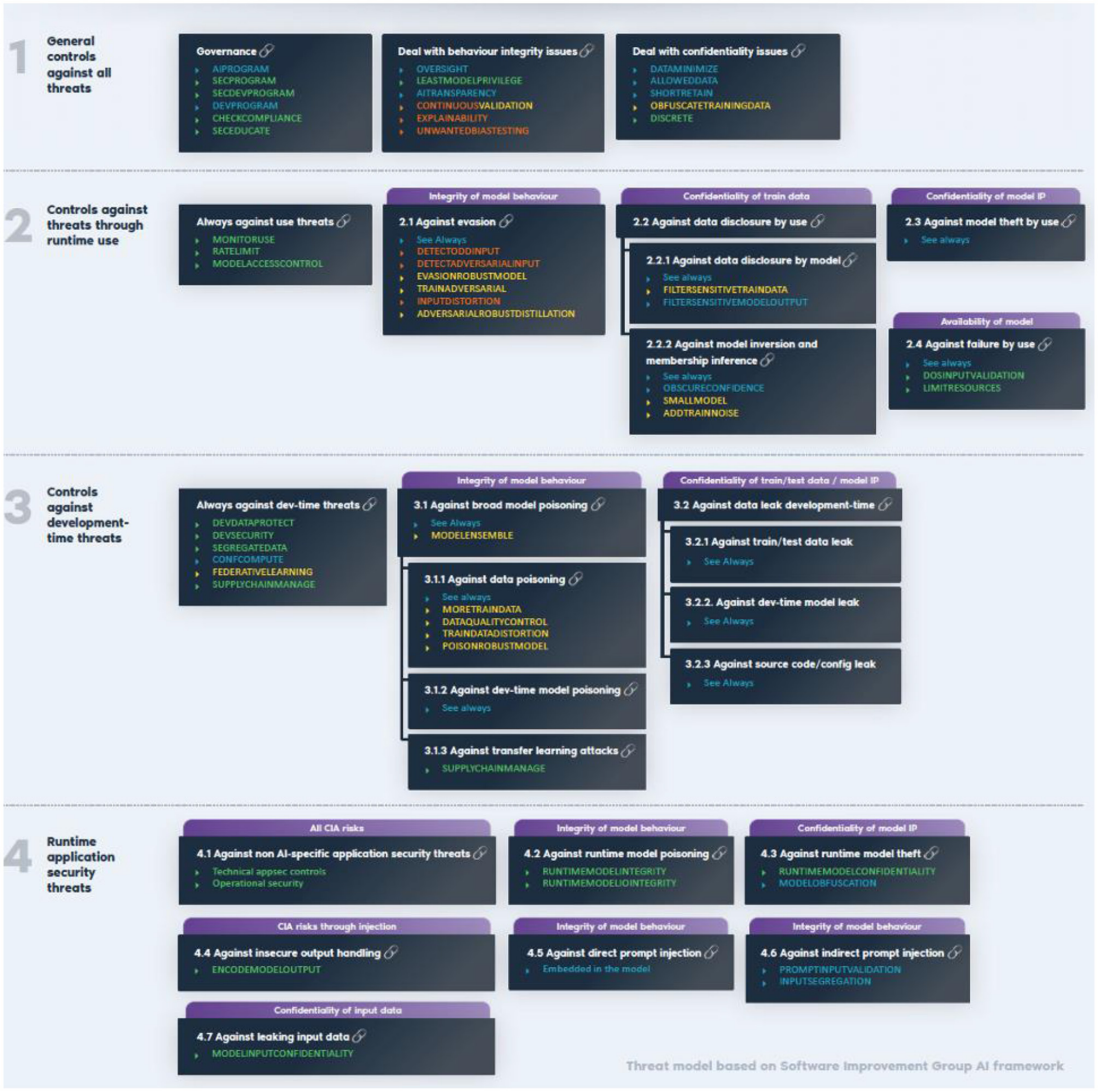
AI security threats and controls navigator (OWASP Top Ten, 2021).

The integration of AI into various facets of society brings forth a new frontier of challenges, particularly in the realm of socio-psychological threats, vulnerabilities, and potential attacks. Understanding and addressing these issues are crucial for the responsible and ethical deployment of AI technologies. Socio-psychological threats in the context of AI often manifest in the form of manipulative tactics and the spread of misinformation. AI algorithms, when applied to social media platforms and information dissemination systems, have the potential to amplify existing biases and polarize communities (Pennycook and Rand, 2018). This can lead to the creation of AI-driven echo chambers that reinforce individuals' pre-existing beliefs, posing a significant threat to social cohesion (see Figure 2). Social threats include:

- **Bias and discrimination:** AI systems may display biases derived from the data they are trained on, potentially resulting in discriminatory outcomes, especially against specific demographic groups.
- **Unfair decision-making:** Automated decision systems can perpetuate existing social inequalities if trained on biased data or if their algorithms lack mechanisms to address fairness.
- **Inequality:** The widespread integration of AI and automation may lead to job displacement, particularly in sectors where routine tasks are easily automated, potentially exacerbating economic inequality. AI algorithms can be challenging to interpret, creating difficulties in holding organizations and systems accountable for their decisions.
- **Misinformation:** AI-generated deepfake content can fabricate realistic but false videos, audio recordings, or images, causing misinformation and potential harm to individuals or reputations. AI algorithms on social media platforms can magnify specific content, contributing to the dissemination of misinformation and the formation of echo chambers.
- **Lack of transparency:** The absence of transparency in AI systems may impede public understanding and trust in how AI is employed.
- **Addiction:** AI algorithms utilized in social media and entertainment platforms can contribute to addictive behaviors, affecting mental health and wellbeing.

AI systems, particularly those employing machine learning algorithms, can be susceptible to vulnerabilities that enable behavioral manipulation. Adversaries may exploit these vulnerabilities to influence user behavior, leading to unintended and potentially harmful consequences. Understanding the psychological aspects of human-AI interaction is crucial in identifying and mitigating such vulnerabilities (Miorandi et al., 2018). The use of AI in data analytics and user profiling raises concerns about privacy erosion. Advanced algorithms can analyze vast amounts of user data, leading to the creation of highly detailed and potentially intrusive user profiles. Unauthorized access to such profiles can result in privacy breaches and compromise the autonomy of individuals (Cavoukian and Jonas, 2017).

AI algorithms, if not carefully designed and monitored, can perpetuate, and amplify existing societal biases. This introduces vulnerabilities in AI systems that may inadvertently discriminate against certain demographic groups. Addressing bias and ensuring fairness in AI decision-making processes are critical to mitigating socio-psychological threats (Diakopoulos, 2016). As AI systems become more integrated into daily life, establishing and maintaining trust between users and AI becomes paramount. Instances of AI errors, biases, or malicious manipulations can erode user trust. Understanding the socio-psychological factors that influence human-AI interaction is essential for developing systems that are not only technically robust but also socially acceptable (Hoffman and Benenson, 2018). The socio-psychological dimensions of AI threats, vulnerabilities, and attacks necessitate a multidisciplinary approach. By addressing these challenges with insights from psychology, sociology, and ethics, we can foster the development of AI systems that enhance societal wellbeing while minimizing potential harms.

## 4 Management of AI trustworthiness

### 4.1 Advanced methodologies for evaluating AI trustworthiness, safety, and risk

The continuous integration of Artificial Intelligence (AI) into critical sectors demands rigorous methodologies for evaluating trustworthiness, safety, and risk. This necessity is driven by the dual aim of harnessing AI's transformative potential while mitigating its inherent vulnerabilities. Among the notable methodologies developed to address these challenges, the S.A.F.E. approach stands out for its holistic assessment framework (Giudici and Raffinetti, 2023). However, while extensively covering information-centric application sectors such as finance, this framework does not seamlessly expand to operation-centric application sectors such as manufacturing, automotive or aerospace, in which greater consideration for AI safety and robustness to adversarial AI threat should be taken. To capture the full spectrum of AI's multifaceted impact, it is essential to explore and integrate additional methodologies that offer specialized insights and solutions. This paper delves into these advanced methodologies, providing a technical and comprehensive analysis.

The S.A.F.E. methodology, emphasizing Security, Accuracy, Fairness, and Explainability, serves as a foundational framework for AI evaluation. It suggests implementing cryptographic safeguards for security, rigorous validation datasets for accuracy, algorithmic audits for fairness, and feature attribution techniques for explainability (Smith et al., 2021). However, the dynamic and complex nature of AI systems requires further methodologies to address emerging challenges. As mentioned in the work of Giudici and Raffinetti (2023) addressing the challenge of ensuring the trustworthiness of AI applications, particularly those based on machine learning (ML) in high-risk areas is of great importance by complying with a set of mandatory requirements such as Sustainability and Fairness. The proposed methodology introduces a set of integrated statistical methods centered around the Lorenz Zonoid tool. This tool is used to assess and monitor whether an AI application is trustworthy over time by measuring key attributes: Sustainability (robustness with respect to anomalous data), Accuracy (predictive accuracy), Fairness (prediction bias across different population groups), and Explainability (human understanding and oversight).

Expanding this methodology to the realm of cyber security and cyber risk management, the principles of S.A.F.E. can be adapted to evaluate and enhance the reliability and integrity of cyber defense mechanisms. In terms of cyber security, the Sustainability metric could help evaluate the robustness of security protocols against evolving threats and attacks, ensuring they remain effective under various scenarios, including extreme conditions such as zero-day exploits or sophisticated cyber espionage activities. Accuracy in this context measures the precision of threat detection systems in identifying genuine threats while minimizing false positives, which is crucial for efficient resource allocation and maintaining operational continuity. Fairness could be applied to ensure that security measures do not unfairly target or exclude any group, maintaining equitable access to digital resources (Barocas and Hardt, 2019; ETSI, 2023). Lastly, Explainability in cyber risk management emphasizes the importance of transparent and understandable security policies and the logic behind automated decisions, such as those made by AI-driven threat detection systems, enabling better stakeholder understanding and trust in cyber security measures. Adopting this SAFE framework in cyber security and risk management can lead to the development of more resilient, fair, and trusted AI-enabled security systems.

An extension to S.A.F.E. is the T.R.U.S.T. framework, which incorporates Transparency, Robustness, Usability, Sustainability, and Traceability. Transparency involves documenting and disclosing AI development processes and data sources. Robustness in machine learning is defined by Goodfellow et al. (2014) as the minimal perturbation required to flip predicted labels. Robustness testing employs adversarial examples and stress tests to evaluate AI resilience (Goodfellow et al., 2014). Usability focuses on the interface between AI systems and users, ensuring that systems are accessible and user-friendly. Sustainability looks at the long-term impacts of AI, including environmental and societal sustainability. Lastly, Traceability ensures that decisions made by AI systems can be tracked to their origin, facilitating accountability.

Worth also mentioning the work that's being done regards evaluation metrics for XAI, organized around objective metrics, such as behavioral, physiological and task performance metrics, and subjective metrics, such as trust and confidence (Zhou et al., 2021). The work of Lopes et al. (2022), proposes a taxonomy for XAI evaluation methods, organized as Human-centered and Computer Centered. Under the human-centered methods some of the evaluation methods are related to the user's perceived system competence and understandability, user's prediction of model output and of model failures. As for the computer-centered, we can find clarity, broadness and simplicity, completeness and soundness.

Mohseni et al. (2021), evaluation metrics are focusing on Mental Models, Explanations Usefulness and Satisfaction, User Trust and Reliance, Human-AI Task performance, and Computational Measures. In Ethical Risk Assessment (ERA) provides a framework for identifying and evaluating ethical risks associated with AI applications. This includes assessing potential biases, privacy breaches, and misuse scenarios. ERA methodologies often involve stakeholder analysis to identify affected parties and ethical impact assessments to evaluate the broader societal implications (Mittelstadt, 2019). These assessments are crucial for developing AI systems that align with ethical norms and societal values.

In the financial sector, Model Risk Management (MRM) methodologies have been adapted to AI, focusing on the accuracy and reliability of models underpinning financial decisions. MRM involves comprehensive testing, including back-testing, scenario analysis, and sensitivity analysis, to ensure that AI models perform as expected across a range of conditions. This rigorous testing is critical for minimizing financial risks and ensuring compliance with regulatory standards (Bank for International Settlements, 2018).

In the aerospace sector, Safety Critical Function Thread Analysis (SCFTA) and Failure Modes and Effects Testing (FMET) methodologies have been applied to enable airworthiness of safety-critical AI applications such as neural flight control systems (Henderson et al., 2022). An AI based system called Maneuvering Characteristics Augmentation System (MCAS) was admittedly involved in the second crash of a BOEING 737 MAX plane in Ethiopia in October 2018, that killed 189 people. Along with technical causes, inadequate pilot training was blamed for the accident. Therefore, this sector shows greater concern for AI safety assurance, human-machine team dynamics and certification methods.

Given the evolving nature of AI systems, continuous monitoring and adaptive risk assessment are paramount. This approach involves real-time monitoring of AI system performance, coupled with adaptive mechanisms to adjust risk assessments based on emerging data. Techniques such as dynamic retraining of models, automatic detection of drift in data distributions, and feedback loops for model adjustment are essential components (Raj and Seamans, 2020).

The evaluation of AI trustworthiness, safety, and risk is a multifaceted challenge that requires a combination of methodologies. While the S.A.F.E. approach provides a solid foundation, the integration of T.R.U.S.T., Ethical Risk Assessment, Model Risk Management, and continuous monitoring offers a more comprehensive and technical framework for assessing AI systems. These methodologies highlight the importance of adaptability, ethical considerations, and rigorous testing in the development and deployment of AI technologies. Future research should focus on refining these frameworks, developing standardized metrics, and fostering interdisciplinary collaboration to ensure the responsible advancement of AI.

As for now, the most usual metrics that we can consider, are described in Table 1.

**Table 1:** Metrics to assess AI properties.

**Property**	**Description**	**Metric**
Accuracy	Amount and frequency of the model's errors, i.e., how “correct” the model's output is compared to reality	Classification Accuracy; Logarithmic Loss; Confusion Matrix; Area under Curve (AUC); F1 Score; Mean Absolute Error; Mean Squared Error
Explainability	Capability of expressing the relation between the feature values of an input to a model and the corresponding output value in a humanly understandable explanation	Coverage; Perceived Understandability; Perceived Technical Competence; Perceived Reliability; Personal Attachment
Fairness	Neutrality of evidence, refers to the property of having models that are not biased by personal preferences, emotions or other limitations introduced by the context	Disparate impact; Differential fairness; Statistical Parity Difference; Demographic Parity Ratio
Reliability	Capability of ML models to maintain a minimum performance level (in terms of accuracy, latency, throughput) under variations in the inputs' distributions	Distance from last best serving ML model or from a benchmark (e.g., rule-based) heuristics; mean time between failure
Robustness	Capability of ML models to operate as expected even under perturbations of the input distributions specifically designed to affect their operation	Misclassification rate, under data perturbation by adversarial samples; evasion success rate (targeted/untargeted evasion attacks)

### 4.2 Technical mitigation measures

Attacks at training stage are generally well-prevented by good MLSecOps practices and conventional risk management methods. Data access can be prevented by appropriate data encryption and access control measures according to conventional cybersecurity standards (e.g., ISO 22600 series). While the use of proprietary data reduces the risk of data access, it does not prevent data leak by authorized insiders. Poisoning attacks can be countered by data sanitization techniques that rely on testing-based identification and removal of examples causing high error rates. Alternatively, robust statistics approaches use constraints and rules to reduce potential distortions of the learning model caused by poisoned data (Biggio and Roli, 2018). AI backdoors are in many cases willingly inserted by software editors for criminal (ransom), commercial (industrial espionage), or strategic purposes (governmental control). Their presence can be revealed by rigorous acceptance testing campaigns (Aufrant and Hervieu, 2020).

Attacks at inference stage must be anticipated, as most palliative measures must be taken at training stage already. Feature squeezing can reduce adversarial perturbations by performing smoothing transformations on input features (Tabassi et al., 2019). Reformers reduce the effect of adversarial perturbations by expanding the properties of a given input to its closest neighbors in the training dataset. The injection of adversarial perturbations in the training data, known as adversarial training, will improve the robustness of a classifier to evasion attacks (Chakraborty et al., 2018). Ensemble methods can reduce the success rate of evasion attacks by combining the results of multiple classifiers based on boosting, bagging or stacking procedures (Biggio and Roli, 2018). Defensive distillation improves model generalizability and robustness by training a distilled NN using knowledge transferred from another NN of similar architecture. Gradient masking techniques can reduce sensitivity to adversarial examples by minimizing the first order derivatives of the model with respect to its inputs (Papernot et al., 2018). Defenses against oracle attacks include Differential Privacy techniques that reduce the risk of model reversing by using general patterns and withholding information about specific individuals in the training procedure (European Defense Agency, 2020). This technique however comes with a cost in terms of model accuracy. An alternative approach is the use of homomorphic encryption that allows machine learning operations on encrypted data. While this technique guarantees privacy protection, it comes with computational expenses and operational limitations (Papernot et al., 2018). Finally, extraction attacks can be detected by the use of model watermarking techniques, by which model answers to specific queries provides formal proof of ownership.

General AI controls were grouped by OWASP and adopted by CEN/CLC JTC21 WG1 TG into four categories: a. General controls against all threats; b. Controls against threats through run time use; c. Controls against development-time threats; d. Controls against runtime application security threats as illustrated in the next OWASP Figure.

### 4.3 Social mitigation measures

Article 9 of the AI Act emphasizes the need for comprehensive risk management in AI systems, necessitating a balance between technical and human oversight. However, both the NIST_AI_RFM (Appendix C) and the ENISA AI Framework underscore the requirement for further research to comprehend the existing limitations of human-AI interaction in the risk management process. A critical aspect yet to be thoroughly explored is the profiling of AI adversaries, specifically focusing on their socio-technical characteristics. This entails delving into the technical skills and social capabilities, including behavioral, ethical, moral, and psychological traits that set the psychological context of an AI attack.

To fortify defense mechanisms against adversarial AI attacks, it is imperative to understand not only the technical aspects but also the social elements influencing such attacks. This encompasses the identification of motives driving attacks, such as financial, commercial, business, or even recreational interests. The defender's effectiveness relies on possessing an appropriate socio-technical profile, understanding the operational priorities of the AI system environment. Early research suggests that integrating socio-technical profiles into security risk assessments enhances the accuracy of estimating social and technical vulnerabilities, enabling a more realistic approach to cybersecurity risk management.

Mitigating social-driven AI threats, such as bias and transparency issues, requires comprehensive measures. Awareness campaigns, social dialogs, and cyber-social experiments are vital components for enhancing the ethical values and principles of both attackers and defenders. Creating digital “clones” of adversarial ML attackers and efficient “question-and-respond” schemes can contribute to the challenge of understanding adversaries better. Investigative psychology research and behavioral science can pave the way for social and behavioral anonymous profiling of AI attackers (Kioskli and Polemi, 2020, 2022a,b).

The introduction of cyber-social exercises provides interdisciplinary training for potential attackers and defenders. This novel approach aims to study attack tactics and defense strategies by considering different social profiles and human characteristics. Developing “clones,” sophisticated ML models inspired by digital twin principles, is crucial for storing attributes and features of adversaries gathered from cyber-social exercises and relevant research. These digital clones, integrated with knowledge graphs and advanced machine-learning techniques, dynamically adapt and respond to evolving threats.

In parallel, a new generation of conversational agents or “chatbots” is proposed to implement intelligent dialogues. These chatbots aim to elicit security operator capabilities in defending AI systems while gathering essential information about the relevant priorities of the business environment. Advances in natural language processing have paved the way for more capable and engaging conversational agents, finding applications across various domains, including business, healthcare, and learning.

Addressing socio-psychological AI threats requires a holistic approach that integrates technical understanding with insights into human behavior. Ongoing research efforts should focus on refining risk management processes, understanding adversary profiles, and developing innovative defense strategies. Recommendations include fostering interdisciplinary collaboration, implementing awareness campaigns, and investing in cyber-social exercises. Moreover, the development of digital clones and conversational agents represents promising directions for advancing AI security. Continuous efforts in these areas will contribute to creating a robust and resilient AI ecosystem that aligns with ethical standards and societal expectations.

## 5 Structuring initiatives for AI trustworthiness

### 5.1 Standards in AI risk management related to trustworthiness

The landscape of standards surrounding AI risks is vast, encompassing contributions from ISO/IEC, ETSI, and IEEE (IEEE P2976, 2021; IEEE P3119, 2021). This section highlights key standards that focus on AI risks and trust management.

Starting with the foundational standards for risk management, such as the ISO27000x series and ISO 31000:2018, we then move to dedicated AI risk management standards like ISO/IEC 24028, which addresses AI security threats.

ISO/IEC 42001—Artificial Intelligence Management System, published in December 2023, is designed to manage risks and opportunities associated with AI, addressing ethics, transparency, reliability, and continuous learning. ISO/IEC 23894 works in conjunction with ISO 31000:2018, focusing specifically on AI risk management. ISO/IEC has also published TR standards, including those that concentrate on AI ethical and societal concerns. The robustness of neural networks is tackled by ISO/IEC 24029-2:2023, which offers a methodology for using formal methods to assess neural network robustness. The development of ISO/IEC 24029-3 aims to focus on statistical methods for this purpose. Technical Report TR 24028 analyzes and surveys approaches to enhance trustworthiness in AI systems and mitigate vulnerabilities related to trustworthiness.

Other relevant ISO standards include:

- ISO/IEC WD 27090—Cybersecurity—Artificial Intelligence: Guidance for addressing security threats to AI systems.
- ISO/IEC WD 27091—Cybersecurity and Privacy: Artificial Intelligence—Privacy protection.
- ISO/IEC 27115—Cybersecurity evaluation of complex systems: Introduction and framework overview.
- ISO/IEC CD TR 27563: Impact of security and privacy in AI use cases.
- ISO/IEC 5338 (also covering the AI risk management process and summarizing 23894).
- ISO/IEC AWI 42105 (under development) on guidance for human oversight of AI systems.
- ISO/IEC 5259 series (Data quality).
- ISO/IEC 24029 series (Robustness).
- ISO/IEC 22989 (AI concept and terminology standard).
- ISO/IEC FDIS 5338: AI system lifecycle processes.

From ETSI, the Securing Artificial Intelligence (SAI) group is making strides in this area. It published the AI Threat Ontology [ETSI GR SAI 001 V1.1.1 (2022-01)] as one of its initial reports. In 2023, ETSI introduced the Artificial Intelligence Computing Platform Security Framework [ETSI GR SAI 009 V1.1.1 (2023-02)], detailing a security framework for AI computing platforms to protect valuable assets like models and data. Additionally, ETSI GR SAI 007 V1.1.1 (2023-03) discusses steps for AI platform designers and implementers to ensure explicability and transparency in AI processing.

IEEE has introduced P3119, a standard for the Procurement of Artificial Intelligence and Automated Decision Systems, establishing definitions and a process model for AI procurement and how government entities can address socio-technical and innovation considerations responsibly. The IEEE P2976—Standard for XAI (eXplainable Artificial Intelligence)—aims to define the requirements for an AI method, algorithm, application, or system to be considered explainable, ensuring clarity and interoperability in AI system design.

In March 2023, the European Commission (EC) requested CEN and CENELEC to work with international and national stakeholders, including SMEs, to develop a European standards program for AI (CEN/CENELEC Standards, 2023). These standards will aim to ensure safety, transparency, user understanding, oversight, accuracy, robustness, cybersecurity, and quality management throughout the AI systems' lifecycle, catering to various stakeholders' needs and ensuring regulatory compliance. This request by the EC was accompanied by a set of requirements in the following areas for the new EU standards:

- **Risk management system for AI systems:** Specifies a continuous iterative process for risk management throughout the AI system's lifecycle, aimed at preventing or minimizing risks to health, safety, or fundamental rights. Ensures integration of risk management systems with existing Union Harmonization legislation where applicable. Drafted for usability by relevant operators and market surveillance authorities.
- **Data and data governance:** Includes specifications for data governance procedures, focusing on data generation, biases, and dataset quality for training AI systems.
- **Record keeping through logging capabilities:** Specifies automatic logging of events for traceability and post-market monitoring of AI systems by providers.
- **Transparency and information to users:** Provides design and development solutions for transparent AI system operations and instructions for users about system capabilities and limitations.
- **Human oversight:** Specifies measures and procedures for human oversight built into AI systems and implemented by users, including those specific to certain AI systems' intended purposes.
- **Accuracy specifications for AI systems:** Lays down specifications for ensuring appropriate accuracy levels, declaring relevant accuracy metrics and tools for measurement.
- **Robustness specifications for AI systems:** Specifies robustness considering potential sources of errors, faults, and interactions with the environment.
- **Cybersecurity specifications for AI systems:** Provides organizational and technical solutions to safeguard AI systems against cyber threats and vulnerabilities.
- **Quality management system for providers of AI systems:** Specifies a quality management system ensuring continuous compliance with various AI system aspects.
- **Conformity assessment for AI systems:** Provides procedures for conformity assessment activities related to AI systems and quality management systems of AI providers.

**Figure 3:**
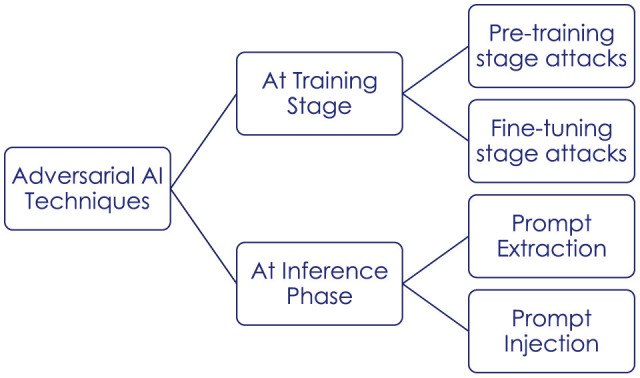
Attacks on LLMs.

Another area of development for standards and methodologies is that of General Purpose AI (GPAI). The release of ChatGPT by OpenAI in December 2022 has triggered a worldwide enthusiasm around consumer-oriented applications of Large Language Model (LLM). Artificial General Intelligence (AGI) techniques with generative capabilities, called foundation models, are being pushed to the market. While these models inherit a lot of the previously highlighted vulnerabilities of narrow AI, their pre-training on a broad set of unlabeled and uncurated data opens a risk of web-scale poisoning and supply chain attacks. Hallucinations, prompt extraction and prompt injection come on top of existing AI risks. In Figure 3 we summarize the main types of attacks on LLMs, which bring additional challenges particularly due to the prompt manipulations.

Existing standards do not appropriately cover the specific risks related with the development and use of GPAI. We would recommend dedicated standardization efforts in this area.

### 5.2 Initiatives and frameworks

In addition to legislation and standards, there several frameworks and research tools that can guide and support the development of AI systems toward their robustness and trustworthiness.

**Figure 4:**
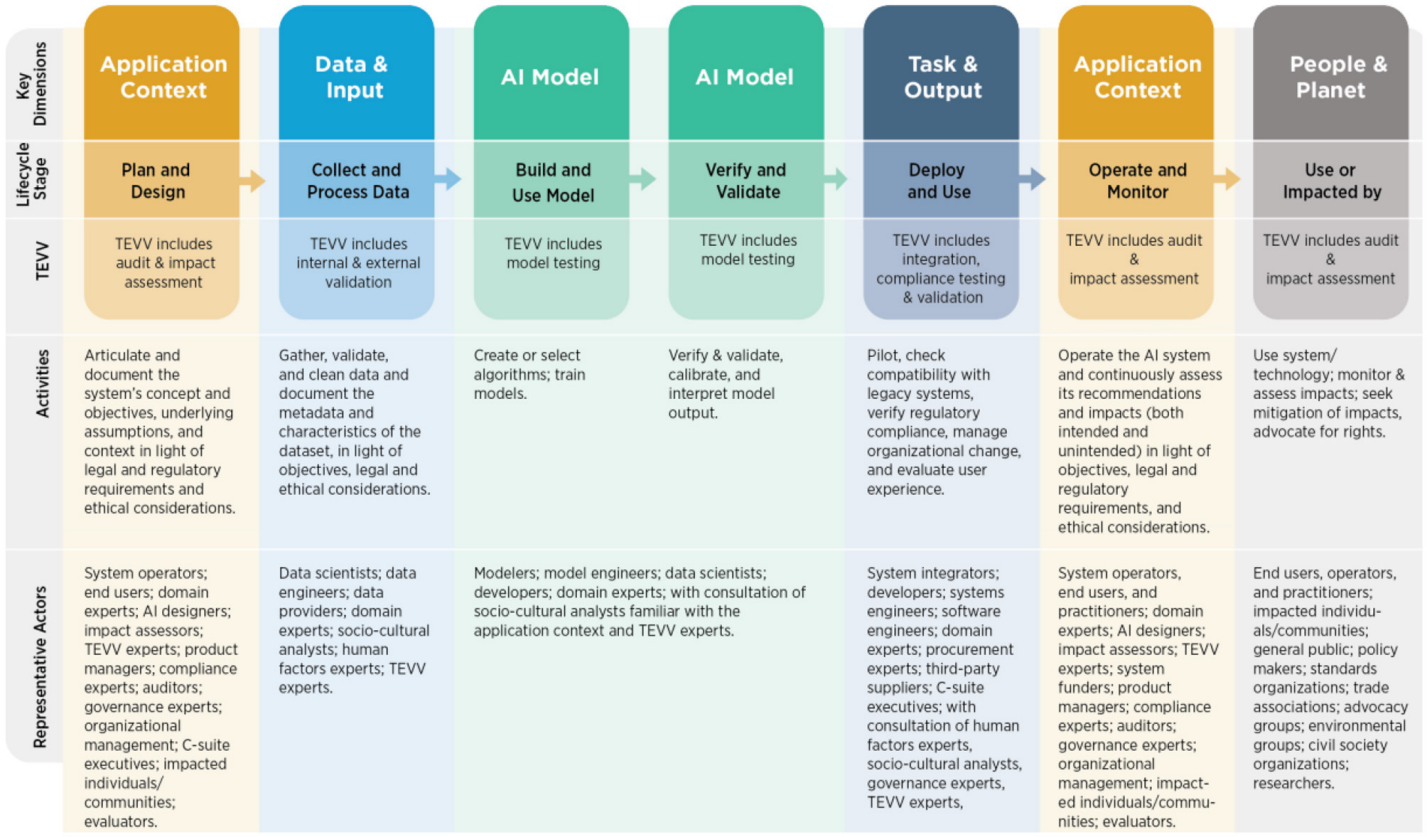
NIST AI RMF AI actors across AI lifecycle stages (NIST AI RMF URL).

NIST AI Risk Management Framework (AI RMF 1.0 URL), consider as characteristic of AI trustworthiness the properties of being valid and reliable, safe, secure and resilient, explainable and interpretable, privacy-enhanced, fair, accountable and transparent. These properties are not seen individually, as they influence each other, and are also tied to the social and organizational behavior, to the decisions made by AI developers and the interactions with the humans. Indeed, the framework assumes that Human judgment is needed to decide on the AI trustworthiness metrics and their thresholds, which are dependent from the decision context. For each of these properties, the framework highlights the risks and how to manage them. The framework core is composed of four functions: Govern, Map, Measure, and Manage. GOVERN is a cross-cutting function that is infused throughout AI risk management and enables the other functions of the process. The MAP function establishes the context to frame risks related to an AI system. The MEASURE function employs quantitative, qualitative, or mixed-method tools, techniques, and methodologies to analyze, assess, benchmark, and monitor AI risk and related impacts. The MANAGE function entails allocating risk resources to mapped and measured risks on a regular basis and as defined by the GOVERN function. Risk treatment comprises plans to respond to, recover from, and communicate about incidents or events. Figure 4 illustrates the actors considered in the framework, along the AI lifecycle.

**Figure 5:**
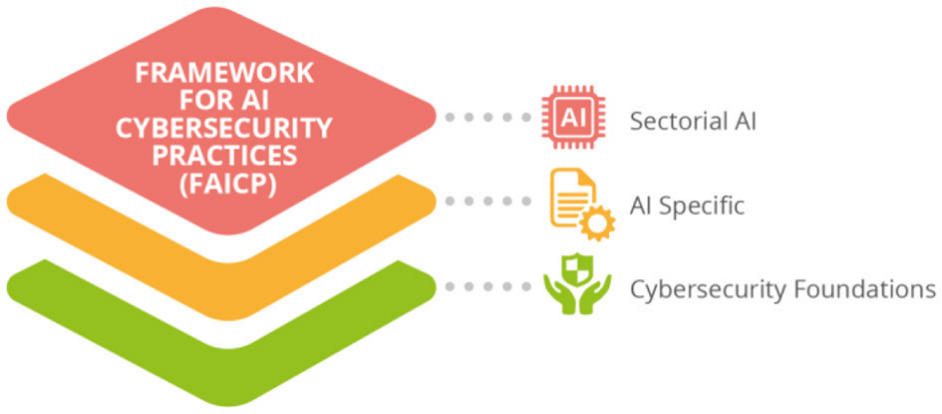
ENISA multilayer framework for AI-related cybersecurity good practices (ENISA URL).

ENISA report on the multilayer framework, provides a vision of good cybersecurity practices for AI based on three layers (see Figure 5): The cybersecurity foundations; AI Fundamentals and Cybersecurity; and Sector Specific Cybersecurity Good practices. The AI layer presents the Ai legislation, describes the assets and procedures, the AI threat assessment and security management, as well as the AI related standards. The standards are mapped along the AI lifecycle of design, development, deploying and monitoring. In the same document, ENISA presents the analyses the current state of cybersecurity requirements and monitoring and enforcement practices that the national competent authorities have adopted or plan to develop. The survey revealed there is still a lot to be done.

**Figure 6:**
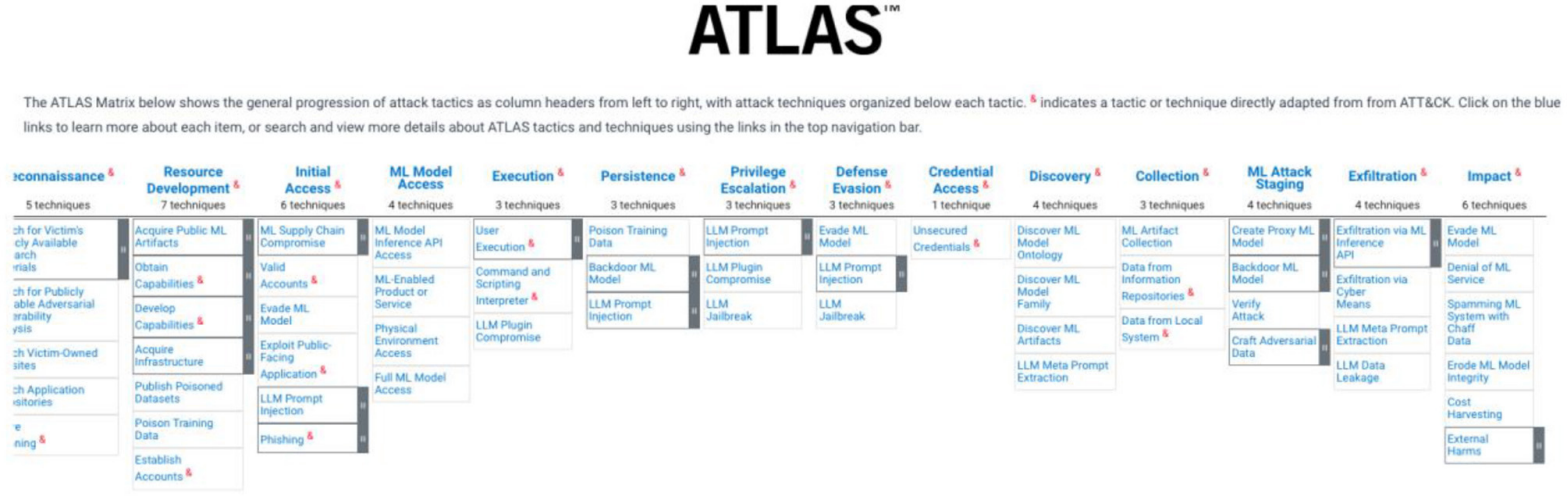
MITRE adversarial threat landscape for AI systems (MITRE ATLAS™ URL).

MITRE ATLAS™ (2024) (Adversarial Threat Landscape for Artificial-Intelligence Systems) provides a knowledge base of adversary tactics and attack techniques on AI systems (see Figure 6). It was modeled after, and is complementary to, MITRE ATT&CK^®^ framework, with some of the tactics and techniques adapted directly from ATT&CK^®^ framework. Here we can find tactics and techniques such as ML Model Access, Privilege Escalation based in Large Language Models (LLM), ML Attack Staging. Worth noticing several attack techniques consider LLMs usage, and for almost all of them there's no mitigation measure mentioned. This is a living knowledge base, strongly recommended to be followed.

ENISA report on the multilayer framework, provides a vision of good cybersecurity practices for AI based on three layers: The cybersecurity foundations; AI Fundamentals and Cybersecurity; and Sector Specific Cybersecurity Good practices. The AI layer presents the Ai legislation, describes the assets and procedures, the AI threat assessment and security management, as well as the AI related standards. The standards are mapped along the AI lifecycle of design, development, deploying and monitoring. In the same document, ENISA presents the analyses the current state of cybersecurity requirements and monitoring and enforcement practices that the national competent authorities have adopted or plan to develop. The survey revealed there is still a lot to be done.

MITRE ATLAS™ (Adversarial Threat Landscape for Artificial-Intelligence Systems) provides a knowledge base of adversary tactics and attack techniques on AI systems. It was modeled after, and is complementary to, MITRE ATT&CK^®^ framework, with some of the tactics and techniques adapted directly from ATT&CK^®^ framework. Here we can find tactics and techniques such as ML Model Access, Privilege Escalation based in LLM, ML Attack Staging. Worth noticing several attack techniques consider LLMs usage, and for almost all of them there's no mitigation measure mentioned. This is a living knowledge base, strongly recommended to be followed.

**Figure 7:**
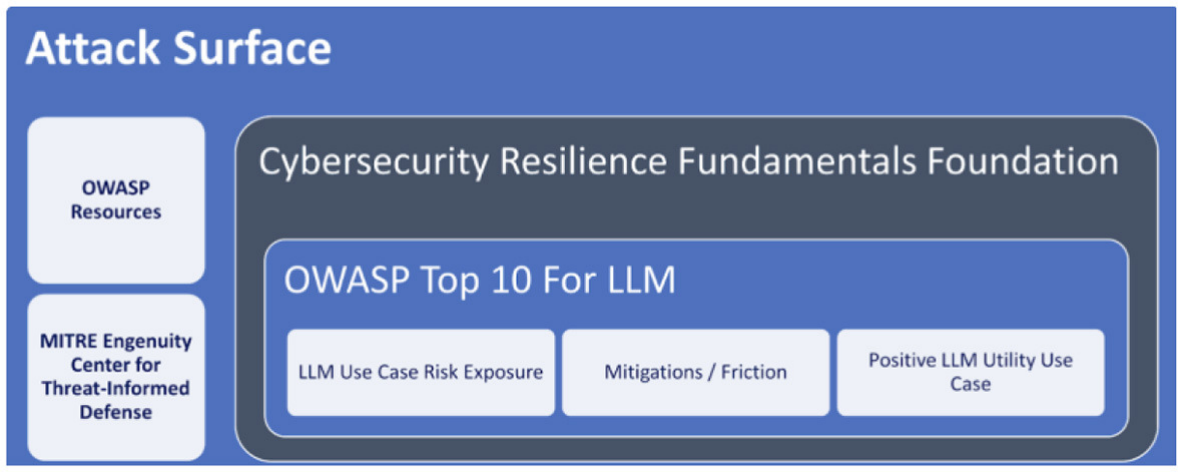
LLM security with OWASP and MITRE resources (OWASP URL).

OWASP is the Open Web Application Security Project that provides the list “OWASP Top 10” (OWASP URL) with the 10 most critical web application security risks (see Figure 7). OWASP includes projects such as ML security Top Ten and the Top 10 for Large Language Model Applications. The ML Security Top 10 addresses the threats mentioned in Section 3.1, such as Data Poisoning, Membership Inference, Transfer Learning Attacks, Model Poising, among others. For each, it provides measures to prevent, risk factors and examples of attack scenarios. OWASP Top 10 for LLM Applications addresses attacks like Prompt Injection, Model Denial of Service, Insecure Plugin Design, among others. The foundation published in December 2023, a document with the essential guidelines for a Chief Information Security Officer (CISO) to manage the rollout of Gen AI technology in their organization.

### 5.3 Research projects and initiatives

In the ever-evolving landscape of AI the imperative for trustworthy and human-centric systems has become paramount. Two pioneering projects funded by the European Union, namely the Fostering Artificial Intelligence Trust for Humans (FAITH) and the Human-centered Trustworthiness Optimization in Hybrid Decision Support (THEMIS 5.0), exemplify dedicated efforts to optimize trust in AI across critical domains. These initiatives not only acknowledge the pressing need for reliable and transparent AI systems but also strive to bridge the gap between technical prowess and human values. In this overview, we delve into the distinctive features of each project, shedding light on their objectives, methodologies, and the broader implications for the future of trustworthy AI. Both FAITH and THEMIS 5.0 serve as exemplars of the EU's commitment to fostering innovation, collaboration, and responsible AI development.

#### 5.3.1 Fostering Artificial Intelligence Trust for Humans (FAITH) project

The FAITH project addresses the increasing demand for trustworthy AI systems across diverse domains during the ongoing digital transformation. Recognizing the critical role AI plays in addressing socio-economic needs, FAITH focuses on optimizing trustworthiness through large-scale pilots in critical domains. Despite existing recommendations and standards, many AI practitioners prioritize system performance over key attributes of trustworthiness. FAITH aims to develop and validate a human-centric trustworthiness optimization ecosystem, focusing on traceability, robustness, security, transparency, and usability. Large-scale pilots will be conducted in critical domains such as robotics, education, media, transport, healthcare, active ageing, and industrial processes. A dynamic risk management approach, following EU legislative instruments and ENISA guidelines, will be employed. The project will deliver widely applicable tools, engage diverse stakeholder communities, and produce sector-specific reports on trustworthiness to accelerate AI adoption. FAITH is an EU-funded innovation action.

#### 5.3.2 Human-centered trustworthiness optimization in hybrid decision support (THEMIS 5.0) project

The THEMIS 5.0 project introduces a cloud-based AI ecosystem designed to optimize trustworthiness in decision support through human-centered approaches. The ecosystem includes AI-driven services engaging with humans through interactive dialogues. A key feature is an AI-driven conversational agent that provides human-interpretable explanations of AI decision-making processes and intelligently elicits knowledge related to decision support needs, moral values, and business goals. THEMIS 5.0 adopts a European human-centric approach, emphasizing co-creation processes to align potential tensions between trustworthy AI components. Co-creation will be applied across eight European countries to ensure widespread acceptability. The project aims to enhance trust in AI systems through transparent and inclusive development. THEMIS 5.0 is an EU-funded initiative.

These research projects contribute significantly to advancing trust in Artificial Intelligence, addressing critical challenges in various application domains. The FAITH project's focus on large-scale pilots and the development of a comprehensive trustworthiness optimization ecosystem provides a holistic approach to AI adoption. On the other hand, THEMIS 5.0 emphasizes human-centered trust optimization in decision support, promoting transparency and inclusivity. To further enhance the impact of these initiatives, it is recommended to foster collaboration and knowledge-sharing between the projects, creating synergies in methodologies, tools, and findings. Additionally, ongoing engagement with diverse stakeholder communities and the incorporation of ethical considerations will contribute to the responsible and ethical deployment of AI technologies. These projects serve as valuable examples of the EU's commitment to promoting trustworthy AI and should inspire future endeavors in the field.

## 6 Conclusions and the ways forward

A comprehensive examination of human factors throughout the entire lifecycle of AI attacks is not merely advantageous but an imperative endeavor. The intrinsic complexity and potential protracted duration of these attacks introduce formidable challenges in terms of detection and monitoring. These complexities render it particularly challenging to discern and integrate credible knowledge concerning the social and behavioral profiles of both attackers and the security operators responsible for defending against these sophisticated threats in the context of risk assessments. Within the realm of assessing trustworthiness, a pivotal facet involves the estimation and development of measurements and scales tailored to social-related threats, such as bias and explainability. This undertaking constitutes a formidable challenge that demands sustained collaboration with experts in social and behavioral sciences. By fostering ongoing partnerships with these specialized professionals, the endeavor is to navigate the intricacies of human behavior intertwined with AI attacks. A key strategy in this pursuit involves the establishment of cyber-social exercises, complemented by face-to-face interviews that engage potential attackers and defenders alike. This concerted approach is not merely an ancillary element but emerges as a critical strategy to effectively address the inherent challenges in assessing and mitigating evolving threats within the AI landscape. Through these collaborative initiatives, the overarching objective is to augment our comprehension of the multifaceted human dimensions associated with AI attacks. Simultaneously, this collective effort seeks to fortify our capacity to assess and proactively mitigate the ever-evolving nature of these threats, thereby ensuring a more resilient and trustworthy AI ecosystem.

## Data availability statement

The original contributions presented in the study are included in the article/supplementary material, further inquiries can be directed to the corresponding author.

## Author contributions

NP: Conceptualization, Formal analysis, Funding acquisition, Methodology, Project administration, Resources, Supervision, Validation, Visualization, Writing – original draft, Writing – review & editing. IP: Formal analysis, Methodology, Validation, Visualization, Writing – review & editing. KK: Formal analysis, Funding acquisition, Investigation, Methodology, Project administration, Resources, Validation, Visualization, Writing – review & editing. AB: Formal analysis, Resources, Validation, Visualization, Writing – review & editing.
